# Learning from experience. Proposal of a refined definition and staging system for bisphosphonate-related osteonecrosis of the jaw (BRONJ)

**DOI:** 10.1111/j.1601-0825.2012.01903.x

**Published:** 2012-02-22

**Authors:** A Bedogni, V Fusco, A Agrillo, G Campisi

**Affiliations:** 1 Section of Oral and Maxillofacial Surgery, Department of Surgery, Azienda Ospedaliera Universitaria IntegrataVerona, Italy; 2 Unit of Oncology, Department of Oncology and Haematology, Azienda Ospedaliera di Alessandria (City Hospital)Alessandria, Italy; 3 Section of Maxillofacial Surgery, Department of Odontostomatologic Sciences, University ‘La Sapienza’Rome, Italy; 4 Sector of Oral Medicine ‘V. Margiotta’, Department of Surgical and Oncological Sciences, University of PalermoPalermo, Italy

Dear Editor,

It is the authors’ belief that the internationally accepted definition of bisphosphonate-related osteonecrosis of the jaws (BRONJ) (Ruggiero *et al*, 2009) has several limitations that prevent clinicians from being confident with the diagnosis of the disease. Following recognition of the non-exposed BRONJ clinical variant (Lazarovici *et al*, 2009), we all became aware that the presence of ‘exposed necrotic bone in the oral cavity’, as outlined in the American Association of Oral and Maxillofacial Surgery (AAOMS) case definition, is just one of the possible clinical manifestations of BRONJ and is not found in all BRONJ patients. As ‘bone exposure’ is certainly not the initial sign of BRONJ in most patients and a minimum of 6–8 weeks’ persistence is required to confirm the clinical suspicion, the final diagnosis is usually delayed for several weeks or months. Therefore, to date, it has been almost impossible to study the early phases of BRONJ. This delayed diagnosis can also explain, at least in part, why the disease is often refractory to the medical and surgical treatments commonly used.

We believe that clinicians will benefit from a definition of BRONJ that contains only robust information, without considering any definite clinical picture or a binding time-frame (i.e. 6–8 weeks). As qualified members of the Expert Panel of the Italian Society for Maxillofacial Surgery (SICMF) and the Italian Society of Oral Pathology and Medicine (SIPMO) on Bisphosphonate-Related Osteonecrosis of the Jaws, we are submitting to the attention of the scientific community the following definition of BRONJ: ‘*bisphosphonate related osteonecrosis of the jaw (BRONJ) is an adverse drug reaction described as the progressive destruction and death of bone that affects the mandible or maxilla of patients exposed to the treatment with nitrogen-containing bisphosphonates, in the absence of a previous radiation treatment*’.

Along with this new definition of BRONJ, we set up and propose a diagnostic work-up to be used to reach the final diagnosis.

**Scheme 1:**
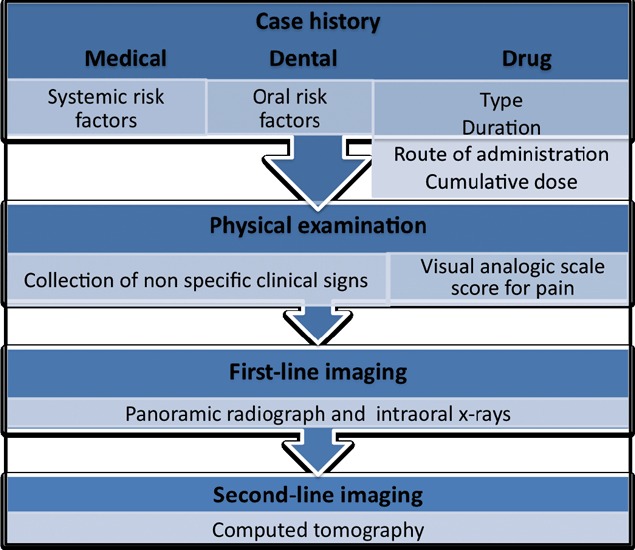
Diagnostic work-up for BRONJ. VAS, visual analogic scale

Although exposed necrotic bone in the oral cavity still remains the best indicator of BRONJ (Ruggiero *et al*, 2009), other non-specific signs and symptoms (Table 1) should raise the suspicion of BRONJ, even in a patient with a well-recognized dental or periodontal disease (Fedele *et al*, 2010). In short, BRONJ should be always investigated in a patient taking nitrogen-containing bisphosphonates (NBP), when one or more clinical signs are present. Because BRONJ is primarily a disease that affects the jawbone, we strongly believe that radiological examination is an important step of the diagnosis. However, as the radiological findings may be characteristic not only of BRONJ (Khan *et al*, 2008), these findings should always match the clinical picture, in order to progress from the clinical suspicion to the final diagnosis. This is of outmost importance as bone biopsies for histology are still not routinely advised for the risk of worsening the disease process. A schematic approach to the work-up for a diagnosis of BRONJ is proposed (Scheme 1), where computed tomography (CT) currently represents the most useful diagnostic tool because of its widespread use and accessibility for patients (Bianchi *et al*, 2007). Structural alteration of trabecular bone is a consistent finding of CT scans performed in patients with BRONJ (Table 2) (Arce *et al*, 2009). CT scans can clearly depict the degree of osteosclerosis of the affected site, visible as the loss of contrast definition between the endosteal cortex and the subjacent medullary bone (i.e. trabecular thickening and/or regional or diffuse osteosclerosis), with respect to the uninvolved bone tissue (Hutchinson *et al*, 2010). Osteosclerosis seems to characterize the early stages of disease and also precedes the occurrence of frank bone exposure in the oral cavity (Saia *et al*, 2010), and thus it should be searched for to provide an early diagnosis.

**Table 1:** Non-specific clinical signs associated with bisphosphonate-related osteonecrosis of the jaws (BRONJ)

*Major clinical sign*
Exposed necrotic bone in the oral cavity
*Minor clinical signs and symptoms*
Abscess
Displaced mandibular stumps
Extra-oral fistula
Gross mandible deformity
Hypoesthesia/paraesthesia of the lips^a^
Mucosal/gengival fistula
Nasal leakage of fluids
Nonhealing postextraction socket
Purulent discharge
Spontaneous expulsion of sequestra and necrotic bone fragments
Sudden dental mobility
Swelling
Toothache and bone pain
Trismus

a Owing to inferior alveolar nerve/infraorbital nerve involvement.

**Table 2:** Non-specific computed tomography (CT) findings associated with bisphosphonate-related osteonecrosis of the jaws (BRONJ)

Early signs	Late signs
Cortical disruption	Diffuse osteosclerosis^b^
Focal bone marrow sclerosis^a^	Oro-antral fistula
Markedly thickened and sclerotic lamina dura	Osteolysis extending to the sinus floor
Persisting alveolar socket	Osteosclerosis of adjacent bones (zygoma, hard palate)
Trabecular thickening^a^	Pathologic fracture
	Periosteal reaction
	Prominence of the inferior alveolar nerve canal
Sequestra formation	Sinusitis

a Density in the sclerotic areas is limited to the alveolar bone region and ranges from little to fully sclerotic with irregular compact and disorganized trabeculation.

b Reduced or complete loss of contrast definition between the endosteal cortex and subjacent medullary bone (density confluence), involving both the alveolar bone and the basal bone of the mandible and maxilla.

Even though magnetic resonance imaging (MRI) provides more detailed information than CT on focal bone marrow alterations (Bisdas *et al*, 2008), the former could provide non-diagnostic results owing to magnetic artifacts caused by the presence of dental casting alloys (Shafiei *et al*, 2003) as well as to motion artifacts caused by the prolonged time necessary to scan the head and neck region (Lenz *et al*, 2000). The majority of BRONJ cases could be diagnosed and staged correctly by the combined use of clinical methods and CT, the latter method currently being more cost-effective and easily accessible than MRI. On the other hand, we believe that MRI should be used to evaluate cases difficult to identify with CT and to establish the real extent of jawbone and soft-tissue involvement in BRONJ patients who are candidates for surgical resection.

The staging system of a bone disease that relies on the use of radiologic imaging for its diagnosis should also be based on the common radiologic features of the disease. The current AAOMS staging system (Ruggiero *et al*, 2009; Migliorati *et al*, 2011), which assigns patients to different stages of disease on the basis almost exclusively of clinical criteria, fails, in our opinion, to consider this important aspect. In this regard, we set up a combined clinical and radiological staging system with the aim of pooling BRONJ patients in different groups also based on the radiological extent of the disease. The staging system we propose (Table 3) differs from the 2009 AAOMS classification as follows:

**Table 3:** Clinical and radiological staging system of bisphosphonate-related osteonecrosis of the jaws (BRONJ)

**Stage 1**	**Focal BRONJ**
	*Clinical signs and symptoms:* bone exposure; sudden dental mobility; nonhealing postextraction socket; mucosal fistula; swelling; abscess formation; trismus; gross mandibular deformity and/or hypoesthesia/paraesthesia of the lips
	*CT findings:* increased bone density limited to the alveolar bone region (trabecular thickening and/or focal osteosclerosis ), with or without the following signs: markedly thickened and sclerotic lamina dura; persisting alveolar socket; and/orcortical disruption
	1a. Asymptomatic
	1b. Symptomatic (pain and purulent discharge)
**Stage 2**	**Diffuse BRONJ**
	*Clinical signs and symptoms:* same as Stage 1
	*CT findings:* increased bone density extended to the basal bone (diffuse osteosclerosis), with or without the following signs: prominence of the inferior alveolar nerve canal; periosteal reaction; sinusitis; sequestra formation; and/or oro-antral fistula
	2a. Asymptomatic
	2b. Symptomatic (pain and purulent discharge)
**Stage 3**	**Complicated BRONJ**
	Same as Stage 2, with one or more of the following:
	*clinical signs and symptoms:* extra-oral fistula; displaced mandibular stumps; nasal leakage of fluids
	*CT findings:* osteosclerosis of adjacent bones (zygoma, hard palate); pathologic mandibular fracture; and/or osteolysis extending to the sinus floor

CT, computed tomography.

1. The absence of Stage 0, so that BRONJ patients with exposed and non-exposed necrotic bone simply represent distinct clinical pictures within the same disease stage.
2. The description of three stages (1–3) based on clinical and CT findings, ‘where Stage 1 includes patients with focal (alveolar bone) osteosclerosis, Stage 2 includes patients with diffuse (alveolar and basal bone) osteosclerosis and Stage 3 includes patients with clinical and radiological signs of advanced and complicated disease.
3. Pain and purulent discharge are no longer used to distinguish between different disease stages. As these symptoms define only asymptomatic (a) and symptomatic (b) forms of BRONJ in patients within the same stage, exclusion of pain and purulent discharge as criteria would limit the ‘ping-pong’ effect (the migration of patients from Stage 1 to Stage 2, and vice versa) that we all experienced when using the AAOMS classification, as a result of the repeated use of antibiotic therapies to treat recurrent bone infections and associated pain.
4. The presence of clinically detectable sequestra is no longer regarded as a sign of complicated disease, as it is for AAOMS Stage 3. In fact, it is a common experience that the spontaneous expulsion or surgical removal of bony sequestra often leads to dramatic, albeit somewhat temporary, clinical improvement, with mucosal epithelialization of the affected site (Ferlito *et al*, 2011).

The definition, work-up and staging system proposed here might, as a whole, provide several practical benefits, in the order: anticipated BRONJ diagnosis and access of patients to therapies; a reduced need for extensive surgical resections with associated long and debilitating postoperative hospitalization; and increased overall efficacy of treatments and patients’ curability. The disadvantage of the additional cost of bone imaging (CT) for the diagnosis would be offset against the overall reduction in treatment costs.

When a new disease is found, little is known of its medical features and behavior; this makes it necessary to avoid general definitions that may include potentially healthy patients (resulting in a higher false-positive risk). This also occurred at the start, when the actual BRONJ case definition was adopted.

At present, with the growing bulk of research on BRONJ, we believe that it is necessary to enter a new phase where clinicians try to establish the initial stages of the disease process, in order to make an earlier diagnosis and improve treatment effectiveness. Although adjustments and updating will be necessary in the future, the definition, work-up and staging system proposed here could achieve this purpose.
